# Remoteness and maternal and child health service utilization in rural Liberia: A population–based survey

**DOI:** 10.7189/jogh.05.020401

**Published:** 2015-12

**Authors:** Avi Kenny, Gaurab Basu, Madeleine Ballard, Thomas Griffiths, Katherine Kentoffio, Jean Bosco Niyonzima, G. Andrew Sechler, Stephen Selinsky, Rajesh R. Panjabi, Mark J. Siedner, John D. Kraemer

**Affiliations:** 1 Last Mile Health, Zwedru, Liberia; 2 Last Mile Health, Boston, MA, USA; 3 Harvard Medical School, Boston, MA, USA; 4 Cambridge Health Alliance, Cambridge, MA, USA; 5 Brigham and Women’s Hospital, Boston, MA, USA; 6 Massachusetts General Hospital, Boston, MA, USA; 7 Georgetown University Medical Center, Washington, DC, USA

## Abstract

**Background:**

This study seeks to understand distance from health facilities as a barrier to maternal and child health service uptake within a rural Liberian population. Better understanding the relationship between distance from health facilities and rural health care utilization is important for post–Ebola health systems reconstruction and for general rural health system planning in sub–Saharan Africa.

**Methods:**

Cluster–sample survey data collected in 2012 in a very rural southeastern Liberian population were analyzed to determine associations between quartiles of GPS–measured distance from the nearest health facility and the odds of maternal (ANC, facility–based delivery, and PNC) and child (deworming and care seeking for ARI, diarrhea, and fever) service use. We estimated associations by fitting simple and multiple logistic regression models, with standard errors adjusted for clustered data.

**Findings:**

Living in the farthest quartile was associated with lower odds of attending 1–or–more ANC checkup (AOR = 0.04, *P* < 0.001), 4–or–more ANC checkups (AOR = 0.13, *P* < 0.001), delivering in a facility (AOR = 0.41, *P* = 0.006), and postnatal care from a health care worker (AOR = 0.44, *P* = 0.009). Children living in all other quartiles had lower odds of seeking facility–based fever care (AOR for fourth quartile = 0.06, *P* < 0.001) than those in the nearest quartile. Children in the fourth quartile were less likely to receive deworming treatment (AOR = 0.16, *P* < 0.001) and less likely (but with only marginal statistical significance) to seek ARI care from a formal HCW (AOR = 0.05, *P* = 0.05). Parents in distant quartiles more often sought ARI and diarrhea care from informal providers.

**Conclusions:**

Within a rural Liberian population, distance is associated with reduced health care uptake. As Liberia rebuilds its health system after Ebola, overcoming geographic disparities, including through further dissemination of providers and greater use of community health workers should be prioritized.

The fragility of health systems in Liberia and neighboring Guinea and Sierra Leone has significantly impeded West Africa’s Ebola Fever Virus (EFV) response [1]. With health systems decimated by civil conflict and an extreme health workforce shortage [2], significant barriers to health care access exist in rural Liberia, where most therapeutic services are delivered from relatively centralized health facilities [3]. These barriers are exacerbated by the EFV outbreak, which, by early December 2014, had killed 175 health care workers—a health workforce disaster that will take years to overcome [4]. Against this backdrop, the country will have to rebuild its health system, with a badly exacerbated health workforce shortage—which will likely be felt most strongly in the most rural areas, where health workers were already most scarce. Better understanding care seeking and utilization in such populations will inform the post–Ebola policy process.

Prior research has found that physical distance from health care facilities is an important determinant of health in resource–limited settings. Both quantitative [5-11] and qualitative [12-14] studies demonstrate that distance, transportation costs, travel time, and attendant opportunity costs disincentivize health care seeking and utilization for a wide range of health conditions, including maternal and child health (MCH) [15].

Relative distance is most often measured bluntly in research and policy planning surveys, largely due to complexities in measurement [16-17]. For example, both Demographic and Health Surveys (DHS) and Multiple Indicator Cluster Surveys dichotomize respondents’ residences as urban or rural. This dichotomization is often translated to national policy and planning documents, which also generally divide populations as rural or urban, and develop strategies and allocate resources based on these categories [18-19]. Such gross categorization prevents more nuanced considerations of remoteness among rural populations, which are typically treated as having homogenous access to health services. It may obscure access issues faced by highly remote populations and lead to policy development that fails to address their needs [20]. More precise distance measurement would enable health officials to better understand distance’s effect on health utilization and mechanisms by which it acts. This, in turn, would enable policymakers to optimize health systems to highly remote populations’ particular needs [21]. As Liberia, Guinea, and Sierra Leone reformulate their health sectors after EFV, such nuance is important for building health systems that are both optimally efficient and effective.

In this study, we aim to estimate the relationship between distance and MCH care seeking and service utilization among a rural Liberian population in the period shortly before EFV’s emergence. We hypothesized that distance to a health facility: 1) impedes care seeking and health service utilization in a dose–response manner, 2) particularly impedes access to services available only available at centralized health facilities, and 3) is associated with greater care seeking from the informal health sector.

## METHODS

### Sampling and survey design

This study analyzed cross–sectional data, originally collected in Konobo and Glio–Twarbo Districts, Liberia, for programmatic purposes to inform the design and implementation of a community health worker (CHW) program. The population sampled represented the target group for the CHW program, who reside in rural districts in southeastern Liberia with an estimated population of approximately 31 000 people and a population density of 12 people per square kilometer [22].

The survey was conducted in August–September 2012, which is during Liberia’s rainy season. We selected households with a two–stage, representative cluster sampling method [23] using 2008 Liberian census data. At the first stage, 30 villages in the two districts were selected randomly with probability proportionate to the overall size of the two districts. We excluded Ziah Town, the only locale meeting Liberia’s definition of an urban area (2000 or more people). We also excluded 25 villages because: 19 had less than 20 households, four could only be reached on foot, and 2 were only accessible by canoe. Together, the excluded villages comprised 15% of Konobo’s rural population. At the second stage, a cluster of 20 households was selected by the following method: 1) spinning a laminated paper triangle on the ground in the village’s center as determined by a map of the village’s extent; 2) using a random number generator to select the first dwelling to survey in the direction indicated by the triangle; and 3) continuing to the next closest dwelling until 20 households were sampled. If no members of a household could be located, the next household was substituted.

The survey’s purpose was to collect demographic, as well as maternal and child health data prior to implementation of a CHW–based maternal and child health program. We surveyed the woman in each household aged 17–and–older who had most recently completed a pregnancy. Women under 17 were excluded because they are considered minors by Liberian national health policies. The survey contained three modules: 1) basic health indicators 2) maternal health questions about the most recent pregnancy and 3) child health. Only participants who had completed a pregnancy within the last five years answered the maternal health module; however, if no woman in the household had completed a pregnancy in the last five years, the other two modules were still administered. The child health module was completed for each of the respondent’s children who were under five and living in the home.

Survey questions were drawn mainly from the 2007 Liberian DHS survey [24]. It was independently translated to Liberian vernacular English by two staff members fluent in the local dialect. Because some participants were expected to speak only Konobo Krahn, a local, non–written language, the survey was administered by bilingual enumerators. All enumerators completed a four–day, pre–study classroom and field training. Following survey administration and entry into a Microsoft Access database, a study supervisor conducted data entry quality assurance by visually checking the first 100 entered surveys. Only one error per 770 fields (0.1% error rate) was identified so the remainder of the surveys were then entered. We also flagged missing and implausible values during data entry and summarization, to request further input from enumerators to clarify and/or update data. Enumerators reported that only one household refused participation.

### Measures

We focused our analysis on maternal and child health care indicators. For maternal health indicators, we selected: 1) one or more antenatal checkups from a health care worker (HCW); 2) four or more antenatal checkups from a HCW; 3) delivery within a health facility attended by any provider; 4) post–natal care (PNC) from a HCW after delivery; and 5) receipt of the full maternal service cascade, defined as at least four ANC checkups, facility–based delivery, and PNC from a HCW. For child health indictors, we selected: care seeking for 1) fever, 2) acute respiratory infection (ARI), and 3) diarrhea if the child experienced those conditions within the two weeks preceding the survey and 4) lifetime receipt of anti–helminthic medication among children over age 1 year. While data were collected on vaccination, we did not include it in this analysis because of low vaccine card possession rates (28%).

Providers were categorized as formal biomedical, informal biomedical, and traditional. Formal biomedical providers were defined as registered facilities or HCWs. Informal biomedical services were those acquired from an informal drug store or mobile drug dispenser. Traditional services were defined as those provided by a traditional healer or the receipt of traditional, herbal medicines. (Provider definitions are provided in **Online Supplementary Document(Online Supplementary Document)**, Table s1.)

For all outcomes, the primary analysis was whether care was sought from a recommended provider: one likely to have appropriate personnel, diagnostic capabilities, and treatments for that condition within this population. For all maternal health services, the recommended care source was a formal biomedical provider. Formal biomedical providers were also the recommended care source for ARI and fever because, consistent with policy, other providers were not trained to accurately diagnose these conditions [25-26]. For diarrhea, the recommended provider was either a formal or informal biomedical provider because both could be expected to carry oral rehydration salts, the recommended diarrhea treatment [27]. For two childhood illnesses, ARI and diarrhea, we also performed analyses to assess care seeking from any source (an indicator of demand for services) and to describe the sources from which care was sought (including multiple provider types) among those who sought care.

The primary predictor variable for all analyses was the road distance from the cluster to Konobo Health Center—the nearest formal health facility, which is located in the district capital, and the only health facility in the study area. Konobo Health Center was able to provide services used as outcome measures (eg, artemesinin combination therapy for fever and oral rehydration solution for diarrhea), and, aside from anti–helminthic treatment, these services generally were not otherwise available at the community level within the formal health care system. Distance was measured with handheld GPS devices (Garmin eTrex 10; Garmin Ltd) by field supervisors during travel to each cluster using recorded GPS tracks. Distance was then divided into quartiles and analyzed as a categorical variable.

We adjusted all analyses for socio–demographic characteristics. For all outcomes, these included maternal age (treated as a continuous variable after assessing appropriate fit using the Box–Tidwell test), current maternal marital status (dichotomous), refugee status (dichotomous), maternal education (categorized as “none,” “primary only,” or “any secondary schooling or higher”), and whether the village is accessible by four–wheel motor vehicles (vs only accessible by bicycle or motorbike). For child health outcome models, we also included child age (dichotomous dummy variables for each year) and gender. Finally, we included whether the cluster was located in a gold mining village (dichotomous), because recent gold discoveries in parts of the surveyed area created population movement with uncertain effects on health service access.

### Statistical methods

Standard summary statistical methods were used to describe respondents’ socio–demographic and clinical characteristics. Differences in descriptive characteristics between distance quartiles were tested using design–corrected chi–squared analysis for categorical variables, and linear regression for normally distributed, continuous variables.

To estimate associations between distance quartiles and the odds of various outcomes, we fit logistic regression models with standard errors adjusted for clustering. For each primary outcome, two models were constructed. First, we fit simple logistic regression models to estimate associations with each predictor. Next, we fit multiple logistic regression models, including all variables identified as potential confounders in prior literature, to identify independent associations with the outcomes of interest. Observations with missing data were excluded, and completeness of data are shown in **Table 1**. Distance quartile was included as set of dummy variables for the main analysis; models were re–run with quartiles as an ordinal variable to test for trends between farther distances and outcomes. After regression, we calculated and graphically depicted the adjusted probability of each outcome using average marginal effects, controlling for all other covariates in the full model at their observed levels.

**Table 1:** Respondents’ socio–demographic characteristics and health conditions by distance quartile

Characteristic	1st quartile (n = 151 households) (n = 147 children)	2nd quartile (n = 163 households) (n = 158 children)	3rd quartile (n = 156 households) (n = 132 children)	4th quartile (n = 130 households) (n = 122 children)	Total (n = 600 households) (n = 559 children)	*P*
**Maternal age, mean (SD)**	37.4 (11.7)	33.8 (9.7)	32.5 (8.9)	32.6 (10.1)	34.1 (10.3)	<0.001
**Married mother**	133/150 (88.7%)	133/163 (81.6%)	129/156 (82.7%)	112/130 (86.2%)	507/599 (84.6%)	0.19
**Refugee**	7/151 (4.6%)	0/163 (0.0%)	5/155 (3.2%)	44/130 (33.9%)	56/599 (9.4%)	<0.001
**Maternal education:**						
none	66/151 (43.7%)	48/161 (29.8%)	48/153 (31.4%)	49/129 (38.0%)	211/594 (35.5%)	<0.001
primary	72/151 (47.7%)	79/161 (49.1%)	76/153 (49.7%)	74/129 (57.4%)	301/594 (50.7%)	
any secondary	13/151 (8.6%)	34/161 (21.1%)	29/153 (19.0%)	6/129 (4.7%)	82/594 (13.8%)	
**Child’s age:**						
<1 year	26/147 (17.7%)	23/158 (14.6%)	32/132 (24.2%)	25/122 (20.5%)	106/559 (19.0%)	0.15
1–5 years	121/147 (82.3%)	135/158 (85.4%)	100/132 (75.8%)	97/122 (79.5%)	453/559 (81.0%)	
**Child’s sex:**						
female	63/147 (42.9%)	86/157 (54.8%)	58/132 (43.9%)	67/122 (54.9%)	274/558 (49.1%)	0.04
male	84/147 (57.1%)	71/157 (45.2%)	74/132 (56.1%)	55/122 (45.1%)	284/558 (50.9%)	
**Pregnant in last 5 years:**	108/151 (71.5%)	120/163 (73.4%)	109/156 (69.9%)	96/130 (73.9%)	433/600 (72.2%)	0.79
one or more ANC checkup	96/103 (93.2%)	99/120 (82.5%)	81/103 (78.6%)	43/96 (44.8%)	319/422 (75.6%)	<0.001
four or more ANC checkups	60/103 (58.3%)	57/120 (47.5%)	48/105 (45.7%)	15/96 (15.6%)	180/424 (42.5%)	<0.001
delivered at a facility*	40/88 (45.5%)	56/107 (52.3%)	62/95 (65.3%)	20/68 (29.4%)	178/358 (49.7%)	<0.001
PNC from health worker*	33/89 (37.1%)	41/107 (38.3%)	40/97 (41.2%)	18/74 (24.3%)	132/367 (36.0%)	0.05
full maternal cascade*	26/90 (28.9%)	29/109 (26.6%)	23/99 (23.2%)	5/76 (6.6%)	83/374 (22.2%)	<0.001
**ARI symptoms:**	38/144 (26.4%)	31/156 (19.9%)	48/129 (37.2%)	31/119 (26.1%)	148/548 (27.0%)	0.05
sought care	24/38 (63.2%)	23/31 (74.2%)	39/48 (81.3%)	21/31 (67.7%)	107/148 (72.3%)	0.28
–formal provider	10/24 (41.7%)	5/23 (21.7%)	2/39 (5.1%)	1/21 (4.8%)	18/107 (16.8%)	0.001
–informal biomedical provider	11/24 (45.8%)	16/23 (70.0%)	31/39 (79.5%)	18/21 (85.7%)	76/107 (71.0%)	0.008
–traditional provider	5/24 (20.8%)	3/23 (13.0%)	5/39 (12.8%)	9/21 (42.9%)	22/107 (20.6%)	0.03
**Diarrhea symptoms:**	47/146 (32.2%)	65/155 (41.9%)	65/130 (50.0%)	54/119 (45.4%)	231/550 (42.0%)	0.03
sought care	20/43 (46.5%)	45/62 (72.6%)	42/62 (67.7%)	26/52 (50.0%)	133/219 (60.7%)	0.01
–formal provider	8/20 (40.0%)	10/45 (22.2%)	0/42 (0.0%)	2/26 (7.7%)	20/133 (15.0%)	<0.001
–informal biomedical provider	13/20 (65.0%)	31/45 (68.9%)	37/42 (88.1%)	18/26 (69.2%)	99/133 (74.4%)	0.12
–traditional provider	1/20 (5.0%)	7/45 (15.6%)	4/42 (9.5%)	15/26 (57.7%)	27/133 (20.3%)	<0.001
**Fever symptoms:**	108/146 (74.0%)	116/155 (74.8%)	101/130 (77.7%)	89/119 (74.8%)	414/550 (75.3%)	0.92
sought care from health facility	46/108 (42.6%)	28/116 (24.1%)	14/101 (13.9%)	8/89 (9.0%)	96/414 (23.2%)	<0.001
**One or more lifetime dewormings**†	95/114 (83.3%)	106/130 (81.5%)	78/95 (82.1%)	47/90 (52.2%)	326/429 (76.0%)	<0.001

ANC – antenatal care, PNC – postnatal care, ARI – acute respiratory infection

*Excludes all women whose pregnancies were not carried to full term.

†Excludes children under one year of age, who are not eligible for deworming.

As a robustness check, we fit the same multivariable models, but excluded refugees and villages with gold mining activities. These populations are the most likely to have moved into or between villages recently, introducing a risk of bias from the possibility that events occurred prior to moving into the study area. Through secondary analyses excluding these populations, the main analyses’ sensitivity to this risk can be assessed.

All statistical analyses accounted for the clustered nature of the data using Taylor linearized variance estimation to adjust standard errors. For maternal health outcomes, data were treated as clustered at the village level. Child health outcomes were further clustered at the household level. We used Stata version 13.1 (StataCorp, College Station, TX) for all analyses. Data were analyzed in 2014.

Use of these data for research purposes was approved by the ethics review boards at the Liberian Institute for Biomedical Research and Partners Healthcare at Harvard.

## RESULTS

Six hundred women completed the survey. Four hundred thirty–three (72.2%) reported a pregnancy in the previous five years. The median distance to the nearest health facility was 28.9 km (km) (range 3.5–50.2 km, **Table 1**) and the median distance to the health facility in each quartile was 10.6, 22.1, 32.3, and 46.3 km, respectively. Maternal respondents’ median age was 34.1 years (interquartile range [IQR] 26–40). Only 2.8% of respondents completed secondary school, and more than one–third (35.5%) received no formal schooling. 9.4% of respondents were refugees from Cote d’Ivoire.

Among women who reported a pregnancy in the past five years, 75.6% attended the ANC at least once, but under half (42.5%) completed four or more visits (**Table 1**). Among respondents who carried a pregnancy to full–term, 49.7% delivered in a health facility and 35.6% received PNC from a HCW. Only 22.2% of respondents received the full cascade of maternal services.

The median number of children younger than five years per household was 1 (IQR 1–2). One hundred six (19%) of the children were infants, and 274 (49.1%) were female. ARI, fever, and diarrhea symptoms were reported in the past two weeks for 148 (27.0%), 414 (75.3%), and 231 (42.0%) children, respectively. Among children with ARI symptoms, families sought care for 72.3%, but only 17.1% sought care from formal medical providers (**Table 1**). Among children with diarrheal symptoms, care was sought for 60.7%, including 50.2% who sought care from a recommended provider. Among those with fever symptoms, only 23.2% sought care from a health facility. Lastly, 76.0% of children over age one year had received anti–helminthic medicine at least once.

**Figure 1:**
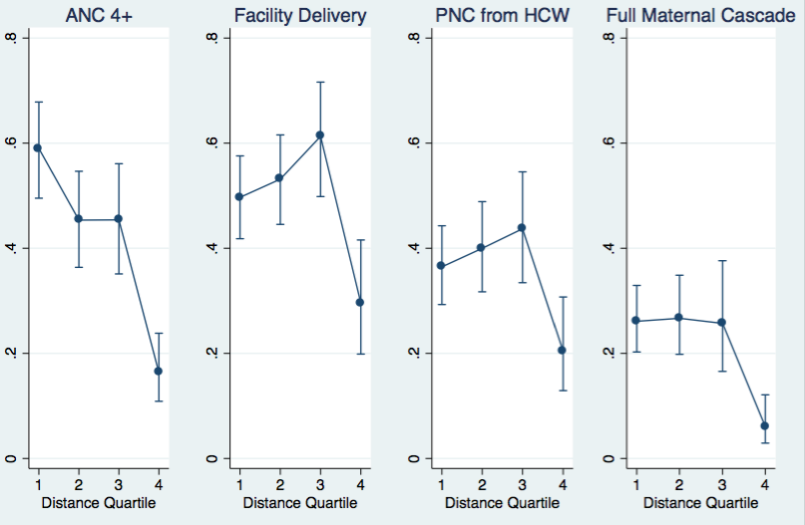
Adjusted probability of maternal care by distance quartile.

We found strong inverse relationships between distance to the nearest health facility and maternal health services uptake (**Table 2**, **Figure 1**). In both the univariable (presented in the **Online Supplementary Document(Online Supplementary Document)**, Tables s2–s3) and multivariable models, women in the fourth distance quartile were significantly less likely than women in the first quartile to attend the ANC at least once (AOR = 0.04, *P* < 0.001) or four times (AOR = 0.13, *P* < 0.001) and the ordinal trend across quartiles of distance to the health facility was significant for both (*P* < 0.001 for both). Women at farther distances were also less likely to access other services, including facility–based delivery (AOR = 0.41, *P* = 0.006 for the most distant vs nearest quartile; *P* = 0.04 for trend), PNC from a HCW (AOR = 0.44, *P* = 0.009 for the most distant quartile; *P* = 0.04 for trend), and complete the full maternal cascade (AOR = 0.18, *P* < 0.001 for the most distant quartile; *P* = 0.001 for trend)

**Table 2:** Maternal health service utilization (full model)

	Antenatal clinic visit 1+		Antenatal clinic visit 4+		Facility–based delivery		Pnc from a formal health care worker		Full maternal cascade	
	**Odds ratio (95% CI)**	***P***	**Odds ratio (95% CI)**	***P***	**Odds ratio (95% CI)**	***P***	**Odds ratio (95% CI)**	***P***	**Odds ratio (95% CI)**	***P***
**Distance quartile:**										
closest	Ref.	Ref.†	Ref.	Ref.†	Ref.	Ref.*	Ref.	Ref.*	Ref.	Ref. †
second	0.29 (0.13–0.67)	0.005	0.57 (0.32–1.00)	0.05	1.15 (0.72–1.84)	0.53	1.17 (0.73–1.87)	0.51	1.03 (0.62–1.70)	0.90
third	0.23 (0.08–0.66)	0.008	0.57 (0.30–1.08)	0.08	1.63 (0.89–3.00)	0.11	1.37 (0.72–2.58)	0.32	0.98 (0.47–2.06)	0.96
farthest	0.04 (0.02–0.09)	<0.001	0.13 (0.07–0.23)	<0.001	0.41 (0.22–0.76)	0.006	0.44 (0.24–0.80)	0.009	0.18 (0.08–0.40)	<0.001
**Moto path**	1.97 (0.99–3.91)	0.05	1.12 (0.70–1.79)	0.62	1.26 (0.74–2.15)	0.38	0.81 (0.50–1.30)	0.37	1.01 (0.61–1.67)	0.96
**Gold mining village**	0.67 (0.31–1.43)	0.31	0.87 (0.50–1.53)	0.63	1.26 (0.70–2.27)	0.44	0.88 (0.48–1.60)	0.66	0.62 (0.32–1.22)	0.16
**Maternal age**	0.98 (0.96–1.00)	0.09	1.02 (0.99–1.04)	0.16	0.98 (0.96–1.01)	0.12	1.00 (0.98–1.03)	0.86	1.00 (0.97–1.03)	0.99
**Married mother**	1.39 (0.73–2.64)	0.30	1.74 (0.94–3.21)	0.08	1.32 (0.75–2.33)	0.32	1.85 (1.07–3.21)	0.03	1.44 (0.76–2.71)	0.25
**Refugee**	2.63 (1.43–4.81)	0.003	1.15 (0.56–2.37)	0.69	1.83 (0.89–3.75)	0.10	2.07 (0.98–4.38)	0.06	1.39 (0.59–3.29)	0.44
**Maternal education:**										
none	Ref.	Ref.	Ref.	Ref.	Ref.	Ref.	Ref.	Ref.	Ref.	Ref.
primary	2.06 (1.31–3.24)	0.003	1.89 (1.26–2.86)	0.003	1.20 (0.79–1.83)	0.37	1.20 (0.78–1.85)	0.40	1.03 (0.67–1.59)	0.89
any secondary	1.53 (0.74–3.16)	0.24	2.28 (1.17–4.44)	0.02	1.85 (0.92–3.72)	0.08	1.38 (0.70–2.74)	0.34	1.36 (0.68–2.72)	0.37

PNC – postnatal care

*P ≤ 0.05, †*P* ≤ 0.001, test for trend across distance quartiles.

**Figure 2:**
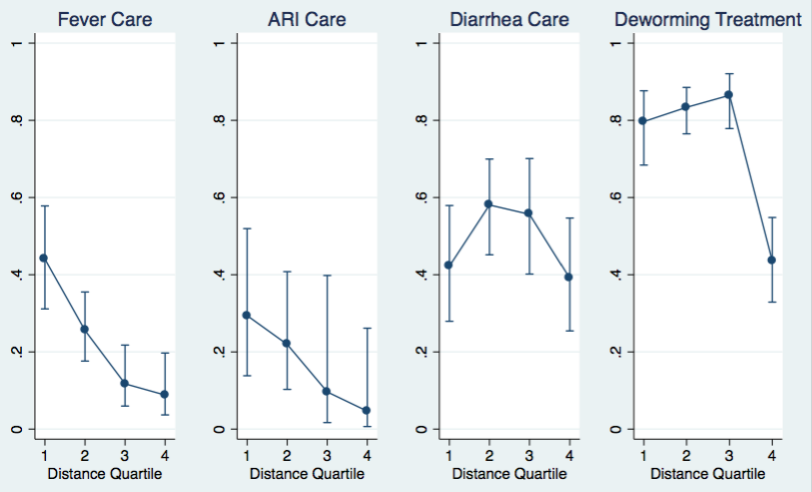
Adjusted probability of child health care seeking or receipt from recommended providers by distance quartile.

Distance from the nearest health facility was also associated with decreased odds of health care seeking for most child health indicators. Odds of anti–helminthic treatment were lower in the fourth distance quartile (AOR = 0.16, *P* < 0.001; *P* = 0.001 for trend across distance quartiles). (**Table 3**, **Figure 2**). For ARI, care seeking from a recommended provider (a health facility) was significantly lower in the third (OR = 0.08, *P* = 0.004) and fourth (OR = 0.07, *P* = 0.01) quartiles in the univariable model, marginally lower in the fourth quartile (AOR = 0.05, *P* = 0.05) in the full model, and marginally significant for trend across distance quartiles (*P* = 0.06). For fever, the odds were lower at all quartiles compared to the closest (*P* < 0.001 for trend; AOR = 0.06 and *P* < 0.001 comparing the fourth to the first quartile). There was no significant relationship between distance and care seeking from a recommended provider (a health facility or informal biomedical provider) for diarrhea.

**Table 3:** Child health care seeking from a recommended provider (full model)

	Fever care seeking from facility		ARI care seeking from formal HCW		Diarrhea care seeking from formal or informal biomedical provider		Deworming treatment	
	**Odds ratio (95% CI)**	***P***	**Odds ratio (95% CI)**	***P***	**Odds ratio (95% CI)**	***P***	**Odds ratio (95% CI)**	***P***
**Distance quartile:**								
closest	Ref.	Ref.*	Ref.	Ref.	Ref.	Ref.	Ref.	Ref.*
second	0.40 (0.18–0.89)	0.03	0.55 (0.10–3.10)	0.49	2.01 (0.86–4.71)	0.11	1.31 (0.60–2.85)	0.49
third	0.15 (0.05–0.43)	0.001	0.14 (0.00–5.43)	0.28	1.80 (0.64–5.10)	0.26	1.70 (0.66–4.40)	0.26
farthest	0.06 (0.03–0.33)	<0.001	0.05 (0.00–1.02)	0.05	0.87 (0.35–2.17)	0.76	0.16 (0.07–0.38)	<0.001
**Moto path**	0.56 (0.25–1.22)	0.14	4.35 (0.66–28.81)	0.12	1.31 (0.57–3.02)	0.51	0.47 (0.21–1.07)	0.07
**Gold mining village**	1.66 (0.69–3.98)	0.24	0.01 (0.00–0.09)	<0.001	0.85 (0.36–1.98)	0.69	0.87 (0.41–1.84)	0.70
**Maternal age**	0.96 (0.92–1.00)	0.04	0.92 (0.75–1.12)	0.39	1.03 (0.98–1.07)	0.27	1.03 (1.00–1.06)	0.07
**Married mother**	0.27 (0.11–0.69)	0.008	0.40 (0.04–3.65)	0.41	0.92 (0.40–2.11)	0.84	1.20 (0.54–2.67)	0.65
**Refugee**	0.54 (0.21–1.37)	0.19	7.85 (0.81–76.02)	0.07	0.79 (0.26–2.41)	0.67	1.43 (0.49–4.14)	0.50
**Maternal education:**								
none	Ref.	Ref.	Ref.	Ref.	Ref.	Ref.	Ref.	Ref.
primary	0.50 (0.26–0.98)	0.04	0.87 (0.15–4.963)	0.87	2.62 (1.25–5.52)	0.01	1.68 (0.97–2.92)	0.06
any secondary	0.51 (0.19–1.37)	0.17	9.63 (1.11–83.64)	0.04	3.36 (1.14–9.88)	0.03	0.97 (0.45–2.11)	0.94
**Child’s age:**								
<1 year	Ref.	Ref.	Ref.	Ref.	Ref.	Ref.		
1–2 years	3.56 (1.45–8.74)	0.007	0.22 (0.02–2.62)	0.22	2.98 (1.29–6.93)	0.01	Ref.	Ref.
2–3 years	3.00 (1.17–7.71)	0.02	0.13 (0.02–0.99)	0.05	1.53 (0.60–3.87)	0.36	1.94 (1.01–3.74)	0.05
3–4 years	3.02 (1.20–7.57)	0.02	0.39 (0.07–2.13)	0.27	2.62 (1.03–6.57)	0.04	2.95 (1.43–6.07)	0.005
4–5 years	1.76 (0.71–4.36)	0.21	0.22 (0.03–1.47)	0.11	1.22 (0.51–2.90)	0.64	2.63 (1.32–5.21)	0.007
**Female child**	1.01 (0.60–1.68)	0.98	0.25 (0.05–1.31)	0.10	0.93 (0.51–1.70)	0.80	1.09 (0.65–1.81)	0.74

ARI – acute respiratory infection, HCW – health care worker

*P ≤ 0.001, test for trend across distance quartiles

In the robustness checks (Online Supplementary Document(Online Supplementary Document), Tables s4–s5), excluding populations most likely to be more highly mobile did not substantially change the relationship between distance quartiles and any health care outcome.

**Figure 3:**
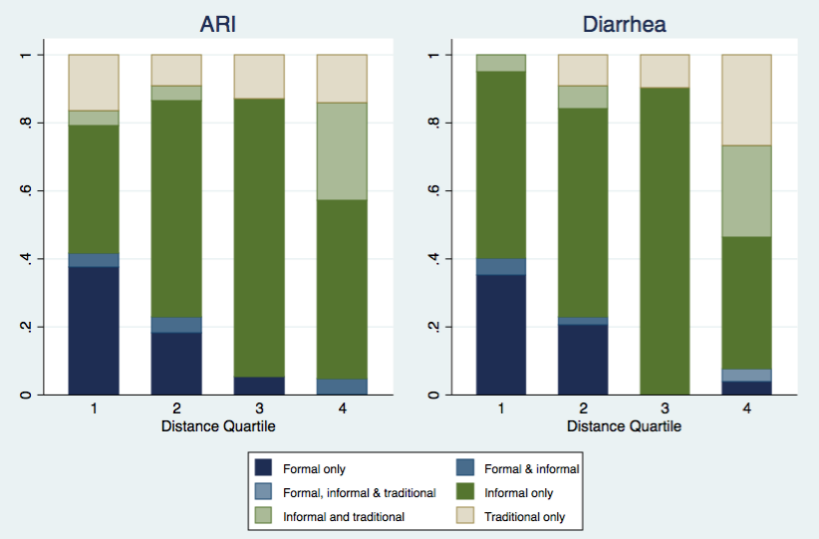
Care sources for ARI and diarrhea among those who sought care by distance quartile.

We also found significant relationships between distance to health care facility and choice of health care provider sought. (**Table 1**, **Figure 3**). For children with ARI symptoms, parents chose care from the formal biomedical sector less often (*P* = 0.001) as distance increased. Care seeking from traditional providers was also more common in the fourth quartile (*P* = 0.03). Similar patterns were observed for children with diarrhea, for which care was less often sought in the formal biomedical sector as distance increased (*P* < 0.001) and utilization of traditional providers increased in the fourth quartile (*P* < 0.001).

## DISCUSSION

Greater distance from facilities is significantly associated with reduced care seeking and service utilization among the rural populations of two districts in southeastern Liberia for several high–priority maternal and child health services. Our estimates for associations between distance to health facilities and service utilization were consistent across multiple health indicators, after adjustment for predicted confounders, and frequently of staggering magnitude. The association between distance and low utilization was particularly strong for services that, in rural Liberia, were only available at facilities or through centralized campaigns (eg, in–facility delivery and deworming). Importantly, dichotomizing this population using rural or urban categorization, as done commonly by DHS and national health ministries, would have resulted in a single homogenous “rural” risk approximation and failed to detect these large differences in health access. Because the outcomes evaluated were among the greatest public health priorities related to Millennium Development Goals, if our results represent other populations in rural Liberia and comparably remote populations elsewhere, they suggest a need to pay greater attention to remoteness both when measuring outcomes and when planning public health programs. .

Our results are consistent with other findings elsewhere in Liberia. Kruk and colleagues found an association between increased travel time and lower utilization of facility–based interventions in northern Liberia [28]. Similarly, Garland et al. found transport difficulties to be a significant barrier to facility–based delivery in north–central Liberia [29]. While national DHS data also generally find care seeking and service utilization to be lower in rural than urban populations [24, 30], analyses of the effect of remoteness *within* rural settings are rarely conducted. Because our population, while very rural, is comparably remote and has similarly limited transport infrastructure to many rural Liberian settings, our findings are likely generalizable to other highly rural Liberian settings.

These findings are also broadly consistent with other data from sub–Saharan Africa. Studies in Burkina Faso [11], Ghana [31], Mozambique [32], Kenya [12], Tanzania [33], and Uganda [16] have documented an adverse association between distance and many outcomes, including service utilization, vaccination, HIV clinic absenteeism, and child mortality. In contrast, a Sierra Leonean study found no association between distance and care seeking for fever, ARI, and diarrhea [34].

With the exception of one rural Kenyan study [12], other research on the effect of distance finds weaker associations than the present study. There are several likely explanations for this. First, this population was more remote than most that have been previously studied and has, as do many highly rural areas, very poor road quality and transportation access. Measurement during the rainy season may have exacerbated these issues. However, although highly remote populations are rarely study populations, they are fairly common in many countries across the continent. Second, objectively measured distances are often dichotomized into closer and farther populations [16, 35], which obscures the effect of farther distances. Finally, many studies use self–reported distance or travel time, which frequently suffer from imprecision^16^ and would likely bias the measured association toward the null.

This paper’s second significant finding is that farther distance is associated with greater informal and traditional provider use. For children with both diarrhea and ARI symptoms, distance did not decrease the likelihood care was sought from *any* provider, but it did markedly affect what type of provider was chosen. Interestingly, the distribution of care providers was similar for diarrhea and ARI even though the recommended providers are different. While this type of provider substitution has not been extensively studied, it is consistent with data from other West African settings [28, 34]. Use of informal health care services is particularly concerning for children with ARI symptoms because substantial evidence demonstrates that failure to accurately diagnosis and provide quality assured therapy confers poor outcomes for this condition [24]. For diarrhea, ORS and zinc are recommended treatment [27]. ORS is regularly available through informal providers, such as mobile drug dispensers and local pharmacies, and zinc often is. While formal sector care would be preferable for diarrhea, higher rates of informal sector utilization is likely less problematic for diarrhea than ARI.

There are a number of possible explanations for the substitution of services observed in this study. It may demonstrate a preference for traditional providers, or low perception of the utility of formal health care services in communities at the farthest distances, or perceptions of illness severity that correlate with distance. Alternatively, the substitution may reflect a supply problem; people may use those services that are most available in their communities. Finally, care seeking decisions may reflect costs—either for services themselves, which would not be expected to vary substantially across distances, or transportation or other transaction costs, which would likely increase with distance. This study cannot discern between these possible causes, but future research will be valuable to do so.

### Limitations

First, our study population was extremely rural and poor, so the generalizability of findings in this study may be limited to similar contexts. The studied region was more rural than average for rural Liberia, but other locations in the country with similar degrees of remoteness and poor road quality are fairly common. Generalization to elsewhere in rural sub–Saharan Africa must be made with caution, but similar distances to health facilities exist in highly rural populations throughout lower population density areas on the continent. Certainly, however, our findings are unlikely to be generalizable to urban areas (with much shorter distances to health facilities and stronger transport networks) or less impoverished countries (with better access to transportation).

Second, we excluded six villages that were extremely remote, including some that were only accessible by canoe because of safety concerns accessing those villages. We did not believe the inclusion of those data justified risks to enumerators. Because these villages were particularly remote, we would expect that their health care access was worse than in villages we sampled. If this is true, the exclusions would result in an underestimate of reduced care seeking and utilization in the farthest quartile. While this introduces bias, the expected direction is toward the null hypothesis, which does not imperil our main findings.

Third, some survey respondents only spoke Konobo Krhan, which is a non–written language, so enumerators translated questions directly during interviews. The use of a non–written survey can introduce challenges to data quality. We mitigated misinterpretation of questions and responses with bilingual enumerators and pre–study translation training. A similar approach is used for DHS surveys in some settings [36], though future research should explore methods to validate field translation. An alternative approach would have been to exclude participants who spoke only Krahn, which may have introduced selection bias because of socioeconomic differences between local groups.

Fourth, this data are susceptible to standard limitation of cross–sectional surveys. The causality of the associations we identified cannot be directly proved, and are susceptible to unmeasured and residual confounding. We also might misclassify distance to clinic if respondents changed residence between the date of health service delivery and the date of the survey. This misclassification should be minimal for child services, due to the short recall period, but may be more problematic for maternal services. We assessed for this risk in sub–analyses in which we excluded populations most likely to have recently moved: refugees (some of whom may have immigrated during the 2010–2011 Ivorian post–election turmoil) and residents of villages with gold mining operations. We found no substantial differences in our estimates with these populations excluded.

Finally, this study did not include direct measures of income or wealth, which some might consider a limitation because there is often a correlation between ruralness and poverty. They were not included for both technical and theoretical reasons. Income is very difficult to measure accurately in the study setting because almost all of the population are subsistence farmers and receive no cash income. Household wealth, on the other hand, is often measured in similar settings by durable good indices. However, measuring relative wealth in settings that are both very rural and very poor is challenged by a lack of supply for many goods in local villages and limited utility for others—such as mobile telephones, which cannot receive service in most of the study area. Finally, the surveyed population is universally impoverished as an absolute measure, limiting the value of relative wealth measurement.

However, by not including measures of income, we are unable to address interactions between geographic and economic barriers to care. Prior research has identified a range of other barriers to care among rural populations in sub–Saharan Africa, including poverty, socio–cultural factors, and poor quality of health services, many of which are often correlated with distance [37-39]. While examining these interactions was beyond the scope of this study, it is an important area for further study, particularly because policy interventions in this or similar settings will have to optimize interventions to simultaneously overcome multiple barriers to care for the same populations.

### Policy implications

This research has several policy implications for highly rural, low–income settings. Our data demonstrate an important need to measure and report distance to health facilities precisely. Doing so will help identify the most vulnerable populations in such settings, enable more disaggregated health indicators, and augment health policy prioritization. International human rights law [40] and ethical norms [41] oblige health ministries and their development partners to promote equal access to essential health services. Our results suggest that, in order to understand and reduce geographic disparities, countries should collect granular data on distance from health services. Important progress has been made on this front. For example, DHS has begun to do so for most of their survey clusters.

More specifically for Liberia as it rebuilds its health system after EFV, this study suggests that there is a need for nuanced approaches to addressing geographic barriers to health care utilization. Ebola has decimated the health workforce—exacerbating an already dire health workforce shortage—with long–lasting consequences for facility–based care provision. Liberia is currently designing a new health workforce strategy and will likely have to increase its reliance on non–facility–based providers in remote areas.

A number of strategies may be useful in this context, including task–shifting [42]; CHW–based service delivery [43-46]; training, formalization, or partnership with traditional or informal providers [47]; mobile clinics and clinical outreach [48-49]; or cash and other reimbursements for health care seeking and/or transportation costs [50-51]. Enhancing the scope and scale of the country’s existing volunteer CHW network—and increased integration between CHWs and facility–based care may be a high–yield, feasible, and sustainable option as the country builds back its health system from Ebola.
