# Supplementary material for: Remoteness and maternal and child health service utilization in rural Liberia: A population–based survey
Source: J Glob Health. 2015 Jun 25;5(2):020401. doi: 10.7189/jogh.05.020401 (PMC4512264; doi:10.7189/jogh.05.020401)
Supplement: Online Supplementary Document [file jogh-05-020401-s001.pdf]

Online Supplementary Document

Kenny et al. Remoteness and maternal and child health service utilization in rural Liberia: A population–based survey

J Glob Health 2015;5:020401

**Table s1. Healthcare Source Definitions**	
Category	Source
Formal biomedical providers*	Doctor
	Nurse
	Midwife
	Other medical provider at a health facility
Informal biomedical providers	Tablet man or black bagger
	Private pharmacy
Traditional providers	Country doctor or zoe
	Traditional midwife

*At the time of the survey, there were no community health workers active in the surveyed population.

Table s2. Maternal Health Service Utilization: Univariable Analyses

	Antenatal Clinic Visit 1+		Antenatal Clinic Visit 4+		Facility-Based Delivery		PNC from a Formal Healthcare Worker		Full Maternal Cascade	
	Univariable Odds Ratio (95%CI)	p	Univariable Odds Ratio (95% CI)	p	Univariable Odds Ratio (95% CI)	p	Univariable Odds Ratio (95% CI)	p	Univariable Odds Ratio (95% CI)	p
Distance Quartile										
Closest	Ref.	Ref.	Ref.	Ref.	Ref.	Ref.	Ref.	Ref.	Ref.	Ref.
Second	0.34 (0.15-0.78)	0.01	0.65 (0.40-1.04)	0.07	1.32 (0.88-1.98)	0.18	1.05 (0.68-1.64)	0.81	0.89 (0.56-1.41)	0.62
Third	0.27 (0.11-0.64)	0.005	0.60 (0.36-1.00)	0.05	2.25 (1.32-3.86)	0.004	1.19 (0.76-1.87)	0.44	0.74 (0.44-1.26)	0.26
Farthest	0.06 (0.03-0.14)	<0.001	0.13 (0.08-0.23)	<0.001	0.50 (0.29-0.85)	0.01	0.55 (0.30-0.99)	0.05	0.17 (0.08-0.39)	<0.001
Moto path	2.41 (1.35-4.30)	0.004	1.57 (1.04-2.38)	0.03	2.18 (1.32-3.62)	0.004	1.05 (0.68-1.63)	0.82	1.16 (0.73-1.86)	0.52
Gold mining village	1.04 (0.60-1.80)	0.88	1.04 (0.66-1.64)	0.85	2.28 (1.41-3.70)	0.001	1.15 (0.76-1.73)	0.50	0.83 (0.51-1.35)	0.43
Maternal age	0.99 (0.97-1.01)	0.31	1.01 (0.99-1.03)	0.24	0.98 (0.96-1.00)	0.07	1.00 (0.98-1.02)	0.89	1.01 (0.98-1.03)	0.58
Married mother	1.35 (0.79-2.29)	0.26	1.55 (0.93-2.61)	0.09	1.55 (0.92-2.60)	0.10	1.80 (1.08-3.01)	0.03	1.33 (0.74-2.38)	0.32
Refugee	0.64 (0.36-1.12)	0.11	0.43 (0.25-0.77)	0.005	0.94 (0.51-1.75)	0.84	1.22 (0.62-2.40)	0.55	0.56 (0.26-1.22)	0.14
Maternal Education										
None	Ref.	Ref.	Ref.	Ref.	Ref.	Ref.	Ref.	Ref.	Ref.	Ref.
Primary	1.63 (1.11-2.40)	0.01	1.53 (1.02-2.28)	0.04	1.37 (0.93-2.00)	0.11	1.16 (0.78-1.74)	0.45	0.98 (0.63-1.51)	0.92
Any Secondary	1.97 (0.97-4.02)	0.06	2.24 (1.25-4.02)	0.009	2.73 (1.50-4.98)	0.002	1.45 (0.80-2.63)	0.21	1.41 (0.75-2.66)	0.27

Abbreviations. PNC: Postnatal care.

Table s3. Child Health Care Seeking from a Recommended Provider: Univariable Analyses								
	Fever Care Seeking from Facility		ARI Care Seeking from Formal HCW		Diarrhea Care Seeking from Formal or Informal Biomedical Provider		Deworming Treatment	
	Univariable OR (95% CI)	p	Univariable OR (95% CI)	p	Univariable OR (95% CI)	p	Univariable OR (95%CI)	p
Distance Quartile								
Closest	Ref.	Ref.	Ref.	Ref.	Ref.	Ref.	Ref.	Ref.
Second	0.43 (0.21-0.90)	0.03	0.41 (0.12-1.40)	0.15	2.16 (0.99-4.71)	0.05	0.88 (0.43-1.82)	0.73
Third	0.22 (0.09-0.51)	0.001	0.08 (0.01-0.41)	0.004	1.78 (0.77-4.16)	0.17	0.92 (0.41-2.04)	0.83
Farthest	0.13 (0.05-0.38)	<0.001	0.07 (0.01-0.55)	0.01	0.73 (0.32-1.70)	0.46	0.22 (0.11-0.45)	<0.001
Moto path	0.70 (0.35-1.38)	0.29	0.81 (0.25-2.64)	0.72	1.85 (0.95-3.61)	0.07	0.86 (0.49-1.49)	0.57
Gold mining village	0.52 (0.25-1.06)	0.07	0.07 (0.01-0.61)	0.02	1.32 (0.70-2.49)	0.37	0.76 (0.44-1.31)	0.31
Maternal age	1.00 (0.97-1.04)	0.83	1.00 (0.93-1.07)	0.89	1.01 (0.97-1.05)	0.55	1.03 (1.00-1.06)	0.08
Married mother	0.35 (0.12-0.98)	0.05	0.79 (0.15-4.34)	0.78	0.78 (0.38-1.60)	0.49	1.00 (0.50-1.98)	0.99
Refugee	0.43 (0.17-1.10)	0.08	1.08 (0.23-5.00)	0.92	0.46 (0.19-1.10)	0.08	0.53 (0.25-1.11)	0.09
Maternal Education								
None	Ref.	Ref.	Ref.	Ref.	Ref.	Ref.	Ref.	Ref.
Primary	0.66 (0.38-1.15)	0.14	1.25 (0.30-5.19)	0.75	1.90 (0.95-3.81)	0.07	1.42 (0.84-2.39)	0.19
Any Secondary	0.72 (0.31-1.68)	0.44	2.47 (0.50-12.13)	0.25	3.50 (1.27-9.63)	0.02	0.92 (0.44-1.94)	0.83
Child's Age								
<1 years	Ref.	Ref.	Ref.	Ref.	Ref.	Ref.		
1-2 years	2.93 (1.30-6.56)	0.01	0.25 (0.04-1.54)	0.13	3.50 (1.57-7.81)	0.003	Ref.	Ref.
2-3 years	2.92 (1.26-6.75)	0.01	0.61 (0.14-2.73)	0.51	1.67 (0.71-3.96)	0.23	1.75 (0.94-3.27)	0.08
3-4 years	2.90 (1.27-6.59)	0.01	0.55 (0.11-2.81)	0.46	2.24 (0.90-5.56)	0.08	2.25 (1.20-4.24)	0.01
4-5 years	1.70 (0.77-3.74)	0.18	0.69 (0.17-2.80)	0.59	1.27 (0.57-2.82)	0.55	2.16 (1.21-3.84)	0.01
Female child	0.95 (0.62-1.46)	0.81	0.62 (0.23-1.67)	0.33	1.00 (0.59-1.70)	1.00	0.91 (0.58-1.44)	0.69

Abbreviations. ARI: Acute respiratory infection. HCW: Healthcare worker.

Table s4. Maternal Health Service Utilization by Distance Quartile: Robustness Checks

	Antenatal Clinic Visit 1+		Antenatal Clinic Visit 4+		Facility-Based Delivery		PNC from a Formal Healthcare Worker		Full Maternal Cascade	
	Full Model Odds Ratio (95% CI)	p	Full Model Odds Ratio (95% CI)	p	Full Model Odds Ratio (95% CI)	p	Full Model Odds Ratio (95% CI)	p	Full Model Odds Ratio (95% CI)	p
Main Analysis										
Closest	Ref.	Ref.***	Ref.	Ref. ***	Ref.	Ref.*	Ref.	Ref.*	Ref.	Ref. ***
Second	0.29 (0.13-0.67)	0.005	0.57 (0.32-1.00)	0.05	1.15 (0.72-1.84)	0.53	1.17 (0.73-1.87)	0.51	1.03 (0.62-1.70)	0.90
Third	0.23 (0.08-0.66)	0.008	0.57 (0.30-1.08)	0.08	1.63 (0.89-3.00)	0.11	1.37 (0.72-2.58)	0.32	0.98 (0.47-2.06)	0.96
Farthest	0.04 (0.02-0.09)	<0.001	0.13 (0.07-0.23)	<0.001	0.41 (0.22-0.76)	0.006	0.44 (0.24-0.80)	0.009	0.18 (0.08-0.40)	<0.001
Excluding Refugees										
Closest	Ref.	Ref.***	Ref.	Ref. ***	Ref.	Ref.*	Ref.	Ref.*	Ref.	Ref. **
Second	0.30 (0.13-0.69)	0.007	0.55 (0.32-0.97)	0.04	1.10 (0.69-1.78)	0.67	1.15 (0.70-1.87)	0.57	1.05 (0.63-1.76)	0.83
Third	0.24 (0.08-0.72)	0.01	0.59 (0.31-1.10)	0.10	1.58 (0.83-2.99)	0.16	1.45 (0.75-2.80)	0.32	1.07 (0.51-2.23)	0.96
Farthest	0.04 (0.02-0.11)	<0.001	0.08 (0.04-0.17)	<0.001	0.31 (0.15-0.63)	0.002	0.33 (0.15-0.72)	0.007	0.11 (0.04-0.37)	0.001
Excluding Gold Mining Villages										
Closest	Ref.	Ref.***	Ref.	Ref. ***	Ref.	Ref.**	Ref.	Ref.*	Ref.	Ref. ***
Second	0.33 (0.14-0.76)	0.01	0.65 (0.37-1.14)	0.13	1.36 (0.88-2.12)	0.16	1.31 (0.83-2.07)	0.24	1.02 (0.61-1.69)	0.95
Third	0.54 (0.13-2.31)	0.40	0.62 (0.31-1.26)	0.18	1.30 (0.70-2.41)	0.40	1.56 (0.79-3.08)	0.19	1.33 (0.63-2.81)	0.45
Farthest	0.03 (0.01-0.07)	<0.001	0.09 (0.05-0.19)	<0.001	0.31 (0.16-0.61)	0.001	0.34 (0.17-0.68)	0.03	0.14 (0.05-0.36)	<0.001
Excluding Refugees and Gold Mining Villages										
Closest	Ref.	Ref.***	Ref.	Ref. ***	Ref.	Ref.**	Ref.	Ref.**	Ref.	Ref. ***
Second	0.33 (0.14-0.78)	0.01	0.60 (0.35-1.06)	0.08	1.29 (0.83-2.02)	0.25	1.22 (0.76-1.96)	0.39	1.01 (0.60-1.69)	0.98
Third	0.52 (0.12-2.21)	0.35	0.58 (0.29-1.15)	0.12	1.19 (0.63-2.27)	0.58	1.46 (0.73-2.94)	0.28	1.34 (0.63-2.84)	0.43
Farthest	0.03 (0.01-0.07)	<0.001	0.07 (0.03-0.15)	<0.001	0.23 (0.10-0.51)	0.001	0.25 (0.10-0.59)	0.03	0.11 (0.03-0.37)	0.001

Abbreviations. PNC: Postnatal care.
Test for trend across distance quartiles: $*p \le 0.05$ $**p \le 0.01$ $***p \le 0.001$. Models control for all covariates in the main analysis.

Table s5. Child Health Care Seeking from a Recommended Provider: Robustness Checks

	Fever Care Seeking from Facility		ARI Care Seeking from Formal HCW		Diarrhea Care Seeking from Formal or Informal Biomedical Provider		Deworming Treatment	
	Full		Full		Full		Full	
	OR (95% CI)	p	OR (95% CI)	p	OR (95% CI)	p	OR (95% CI)	p
Main Analysis								
Closest	Ref.	Ref.***	Ref.	Ref.	Ref.	Ref.	Ref.	Ref.***
Second	0.40 (0.18-0.89)	0.03	0.55 (0.10-3.10)	0.49	2.01 (0.86-4.71)	0.11	1.31 (0.60-2.85)	0.49
Third	0.15 (0.05-0.43)	0.001	0.14 (0.00-5.43)	0.28	1.80 (0.64-5.10)	0.26	1.70 (0.66-4.40)	0.26
Farthest	0.06 (0.03-0.33)	<0.001	0.05 (0.00-1.02)	0.05	0.87 (0.35-2.17)	0.76	0.16 (0.07-0.38)	<0.001
Excluding Refugees								
Closest	Ref.	Ref.***	Ref.	Ref.	Ref.	Ref.	Ref.	Ref.***
Second	0.40 (0.18-0.90)	0.03	0.63 (0.10-3.93)	0.61	1.75 (0.73-4.21)	0.20	1.25 (0.58-2.68)	0.56
Third	0.13 (0.04-0.42)	0.001	0.17 (0.00-7.17)	0.34	1.45 (0.48-4.45)	0.50	1.82 (0.70-4.75)	0.21
Farthest	0.13 (0.04-0.44)	0.002	0.11 (0.00-3.04)	0.18	0.74 (0.25-2.15)	0.57	0.11 (0.05-0.24)	<0.001
Excluding Gold Mining Villages								
Closest	Ref.	Ref.***	Ref.	Ref.	Ref.	Ref.	Ref.	Ref.***
Second	0.36 (0.16-0.85)	0.02	0.77 (0.13-4.65)	0.77	1.57 (0.64-3.86)	0.31	1.26 (0.56-2.85)	0.56
Third	0.17 (0.04-0.77)	0.02	0.11 (0.00-11.90)	0.34	2.28 (0.53-9.82)	0.26	4.31 (0.67-27.68)	0.12
Farthest	0.12 (0.04-0.38)	0.001	0.05 (0.00-1.30)	0.07	0.64 (0.22-1.92)	0.42	0.14 (0.06-0.31)	<0.001
Excluding Refugees and Gold Mining Villages								
Closest	Ref.	Ref.***	Ref.	Ref.	Ref.	Ref.	Ref.	Ref.***
Second	0.37 (0.16-0.87)	0.02	0.90 (0.13-6.12)	0.91	1.35 (0.54-3.34)	0.51	1.21 (0.55-2.65)	0.62
Third	0.18 (0.04-0.79)	0.03	0.13 (0.00-15.71)	0.39	1.94 (0.43-8.78)	0.38	4.01 (0.57-28.1)	0.16
Farthest	0.13 (0.04-0.46)	0.002	0.11 (0.00-3.46)	0.20	0.40 (0.12-1.35)	0.14	0.10 (0.05-0.22)	<0.001

Abbreviations. ARI: Acute respiratory infection. HCW: Healthcare worker.
Test for trend across distance quartiles: *p ≤ 0.05 **p ≤ 0.01 ***p ≤ 0.001. Models control for all covariates in the main analysis.
